# Society Meetings

**Published:** 1901-11

**Authors:** 


### SOCIETY MEETINGS.

### Buffalo Academy of Medicine.

*Section on Pathology, September 17, 1901.*

Reported by IRVING PHILLIPS LYON, M. D., Secretary

The first regular meeting of the year of the Section on Pathology, Buffalo Academy of Medicine, was held in Alumni Hall, University of Buffalo, September 17, 1901, at 8.30 p.m.

The essayist was Professor Simon Harry Gage of Cornell University, Ithaca, who presented Some demonstrations, with projecting microscope of embryological specimens, including some abnormalities. The paper was discussed by Drs. Williams, Van Peyma and Benedict, and a vote of thanks was tendered Professor Gage for his able address.

### The Buffalo Academy of Medicine.

OFFICERS—SESSIONS 1901-1902.

PRESIDENT, Charles G. Stockton; secretary, Francis W. McGuire; treasurer, Charles S. Jewett; trustees, Matthew D. Mann, three years; Joseph W. Grosvenor, two years; Stephen Y. Howell, one year. The vice-presidents by virtue of being chairmen of the various sections are: Julius Ullman, chairman, section of medicine; Harvey R. Gaylord, chairman, section of surgery; Thomas F. Dwyer, chairman, section of obstetrics and gynecology; Grover W. Wende, chairman, section of pathology; Elmer G. Starr, chairman, section of ophthalmology, otology, laryngology and rhinology. The council is therefore composed of the aforementioned officers, the president of the Academy of the previous year, Marcel Hartwig, and the secretaries of the sections, who are: Albert E. Woehnert, secretary, section of medicine; Herriot C. Rooth, secretary, section of surgery; Wm. H. Mansperger, secretary, section of obstetrics and gynecology; Irving P. Lyon, secretary, section of pathology; Arthur G. Bennett, secretary, section of ophthalmology, otology, etc.

PROGRAM, 1901-1902.

September 3, Surgery—Some points on resection of the hip and knee joints, Herman Mynter.

September 10, Medicine—Stated meeting of Academy. Treatment of pneumonia, DeLancey Rochester. Intestinal dystrypsia, J. C. Hemmeter, Baltimore.

September 16, Ophthalmology, etc.—Methyl alcohol; should it be placed on the list of poisons? Swan M. Burnett, Washington, D.C.

September 17, Pathology—Some demonstrations with projecting microscope of embryological specimens, including some abnormalities, Prof. Simon Harry Gage, Cornell University. Vegetating dermatitis developing during the course of infantile eczema, Herman K. DeGroat.

September 24, Obstetrics—Some experiences in a series of obstetrical cases, Eli H. Long. Diagnosis in vertex presentations, P. W. Van Peyma.

October 1, Surgery—Subject to be announced, C. A. Hamann, Cleveland.

October 8, Medicine—Dyspeptic asthma, Max Einhorn, New York City. Subject to be announced, Allen A. Jones.

October 15, Pathology—Subject to be announced, Frederick C. Busch.

October 21, Ophthalmology, etc.—The treatment of the commonest form of deafness, George F. Cott.

October 22, Obstetrics—Concerning the diagnosis and treatment of the various forms of septic pelvic diseases, Ferdinand Henrotin, Chicago.

November 5, Surgery—Subject to be announced, Eugene A. Smith.

November 12, Medicine—Some facts and fallacies concerning pulmonary tuberculosis, John H. Pryor.

November 18, Ophthalmology, etc.—Stated meeting of Academy. Medical and surgical aspect of the Boer war, George Sterling Ryerson, Toronto.

November 19, Pathology—Diseases in the Philippines, Lewellyn E. Barker.

November 26, Obstetrics—Pelvic lesions in relation to their distinctive effects upon mental disturbances, A. T. Hobbs, London, Ont. Reproduction of original work done in microscopic embryology, accompanied by remarks on heredity, Adele A. Gleason.

December 3, Surgery—Subject to be announced, Charles H. Frazier, Philadelphia.

December 10, Medicine—Clinical night. Little's disease, Irving M. Snow; Friedreich's ataxia, Dewitt H. Sherman.

December 16, Ophthalmology, etc.—The original operation for extraction of cataract as devised by Daviel, Alvin A. Hubbell.

December 17, Pathology—Subject to be announced, William Osler, Baltimore. Subject to be announced, Herbert U. Williams.

December 24, Obstetrics—Peritoneal tuberculosis, William H. Mansperger. Concerning the management of labor cases, Stephen Y. Howell.

January 7, Surgery—The blood and its relation to surgical diagnoses, Albert E. Woehnert.

January 14, Medicine—Subject to be announced, A. L. Benedict. The capillary area, Eli H. Long.

January 20, Ophthalmology, etc.—Subject to be announced, Lucien Howe.

January 21, Pathology.—Relation of evolution to infection with special reference to venereal diseases, G. Frank Lydston, Chicago. Discussion by Roswell Park, William H. Heath and

Walter D. Greene. The pathology of stricture of the urethra, J. Henry Dowd.

January 28, Obstetrics. Stated meeting of Academy. Subject to be announced, William R. Pryor, New York City.

February 4, Surgery—Subject to be announced. Dr. Archibald, Montreal.

February 11, Medicine—Syphilis of the nervous system, Floyd S. Crego. Cardiac asthma, H. L. Elsner, Syracuse.

February 17, Ophthalmology etc—Subject to be announced, Frank W. Hinkel.

February 18, Pathology—Clinical and pathological cases in dermatology (stereopticon), John A. Fordyce, New York City.

February 25, Obstetrics—Subject to be announced, J. Montgomery Baldy, Philadelphia.

March 4, Surgery—Stated meeting of Academy. Subject to be announced, Frederic Kammerer, New York City.

March 11, Medicine—Hysterical joint affections, Prescott LeBreton. The present conception of neurasthenia, H. B. Singer.

March 17, Ophthalmology, etc.—Diseases of skin affecting the nose (stereopticon), Grover W. Wende.

March 18, Pathology—Tropical and amebic dysenteries, Simon Flexner, Philadelphia.

March 25, Obstetrics—Placenta previa, Matthew D. Mann. Ectopic pregnancy, Henry D. Ingraham.

April 1, Surgery—Subjects to be announced, Harvey W. Cushing, Boston, and Roswell Park.

April 8, Medicine—Subject to be announced, Irving M. Snow. Pernicious anemia, Charles G. Stockton, and Albert E. Woehnert.

April 15, Pathology—Some practical applications of physiologic chemistry, A. L. Benedict.

April 21, Ophthalmology, etc.—The conservative treatment of chronic otitis media, Max Keiser.

April 22, Obstetrics—Subject to be announced, Lewis S. McMurtry, Louisville.

May 6, Surgery—Wry neck, Prescott LeBreton. Subject to be announced, Bernard Bartow.

May 13, Medicine—Subject to be announced, J. M. Anders, Philadelphia. Children's form of rheumatoid arthritis, Dewitt H. Sherman.

May 19, Ophthalmology, etc.—Subject and essayist to be announced.

May 20, Pathology—Stated meeting of Academy. Subject to be announced, William H. Welch, Baltimore.

May 27, Obstetrics—Subject to be announced, Charles A. L. Reed, Cincinnati.

---

The 14th International Medical Congress will be held in a year and a half, preparations for which already have begun. The executive committee consists of the President, Professor Julian Callejay Sanchez; secretary, General Dr. Angell Fernandez-Caro y Nouvilas; treasurer, Dr. Jose y Ocma; members, the president and secretaries of the different sections.

The congress will be convened at Madrid, under the protection of His Majesty, King Alphonse xiii., and his august mother, the Queen Regent, from the 23d to 30th of April, 1903. The object of the congress is to be purely scientific.

The cost of registration is 30 pesetas, (about six dollars) and should be paid to the secretary-general, at the Faculty of Medicine, Madrid, before the date of the meeting or at the time of registration, and will permit the holder to all the privileges offered by the congress.

The congress will be divided into the following sections: (1) Anatomy, anthropology, comparative anatomy, embryology, descriptive anatomy, normal histology and teratology; (2) physiology, physics and chemical biology; (3) general pathology; pathological anatomy and bacteriology; (4) therapeutics, pharmacology and materia medica; (5) internal pathology (general medicine); (6) neuropathology, mental diseases and criminal anthropology; (7) pediatrics; (8) dermatology and syphilography; (9) surgery and surgical operations; (10) ophthalmology; (11) otology, rhinology and laryngology; (12) odontology; (13) gynecology and obstetrics; (14) medicine and military and naval hygiene; (15) hygiene, epidemiology and sanitary science; (16) legal medicine.

Ladies accompanying members will have the benefit of all reductions on railroads and may assist at all fêtes and ceremonies given in honor of the members, on payment of 12 pesetos (about $2.25.)

---

The American Electro-Therapeutic Association held its eleventh annual meeting in Buffalo, September 24-26, 1901. Dr. Ernest Wende, of this city, was the president, and during his annual address discussed the advances in the application of electricity to therapeutics, and cited what it had accomplished as an aid to medical science. During the meeting an evening session was held in the New York State building at the exposition, at which an interesting paper was read by Dr. A. W. Bayliss, of Buffalo, and there also was an address by Mr. Henry Rustin, superintendent of the electrical department of the exposition.

---

THE American Public Health Association, including organisations in Canada, Mexico and the United States, held its 29th annual meeting in Buffalo, September 16-20, 1901.

Dr. Benjamin Lee, of Philadelphia, was the president. Among the delegates was Dr. Eduardo Liceaga, of Mexico, who was president of the association when it met in Buffalo four years ago.

At its final session New Orleans was chosen as the place for the meeting next year. The election of officers resulted as follows: President, Henry D. Holton, Brattleboro, Vt.; first vice-president, Walter Reed, U. S. A.; second vice-president, Jesus Chico, Guanajuato, Mexico; secretary, Charles O. Probst, Columbus, O.; treasurer, Frank W. Wright, New Haven, Ct.; members of the executive committee, to serve for two years, Gardner T. Swarts, of Providence, W. C. Gorgas, of Havana, J. C. Schrader, of Iowa; member of the executive committee, to serve for one year, Fernando Lopez, of Mexico.

The committee on public health legislation presented a report through the chairman, U. O. B. Wingate, of Milwaukee. The report dealt mainly with the Spooner bill, which provides for the creation of a bureau in the treasury department to have supervision of matters pertaining to public health. The report was adopted. Several other committees made reports.

---

THE Southern Surgical and Gynecological Association will hold its fourteenth annual meeting at Richmond, Va., November 12, 13 and 14, 1901. The officers are: President, Manning Simons, Charleston; vice-presidents, Geo. H. Noble, Atlanta, and L. C. Bosher, Richmond; secretary, W. D. Haggard, Jr., Nashville; treasurer, Floyd W. McRae, Atlanta; council, Geo. J. Engelman, Boston; Ernest S. Lewis, New Orleans; George Ben Johnston, Richmond; L. McLane Tiffany, Baltimore; Lewis S. McMurtry, Louisville; chairman committee of arrangements, George Ben Johnston, Richmond. A large and excellent program has been prepared. All railroads will give one and one-third rate fare on the certificate plan. Membership blanks may be had upon application. Titles of papers or corrections should be sent at once to W. D. Haggard, Jr., secretary, Nashville, Tennessee.

---

THE Buffalo Academy of Medicine held meetings during the month of October, 1901, as follows:

*Section on Surgery.*—Tuesday evening, October 1. Program: Spasmodic torticollis and its surgical treatment, C. A. Hamann, Cleveland. Discussion opened by Eugene A. Smith and J. W. Putnam.

*Section on Medicine.* Tuesday evening, October 8. Program: Dyspeptic asthma, Max Einhorn, New York City; Recurrent vomiting, Allen A. Jones; discussion opened by Charles G. Stockton and A. L. Benedict.

*Section on Ophthalmology, Otology, Laryngology, and Rhinology.*—Monday evening, October 14. Program: Exophthalmic goiter; its symptoms, diagnosis, pathology and treatment, Casey A. Wood, Chicago; discussed by A. A. Hubbell, W. C. Krauss and J. W. Putnam. The treatment of the commonest forms of deafness, Geo. F. Cott.

*Section on Pathology.*—Tuesday evening, October 15. Program: The present status of our knowledge of the innervation of the heart, Frederick C. Busch. Vegetating dermatitis developing during the course of infantile eczema, Herman K. De Groat.

---

AT THE 27th annual meeting of the Mississippi Valley Medical Association held at Put-in-Bay, the following officers were elected for the ensuing year: President, S. P. Collins, Hot Springs, Ark.; first vice-president, J. C. Culbertson, Cincinnati; second vice-president, Paul Paquin, Asheville; secretary, Henry Enos Tuley, Louisville; treasurer, Thos. Hunt Stucky, Louisville; chairman committee of arrangements, A. H. Cordier, Kansas City. The next meeting is at Kansas City, Mo., October, 1902.

---

THE Erie Railway Surgeons Association held its tenth annual meeting at Buffalo, October 16, 1901, under the presidency of

Dr. W. L. Cuddeback, of Port Jervis. The program announced ten scientific papers with discussions; the president's address on The railroad company, the employee and the railroad surgeon, their mutual relation; and a clinic by Dr. C. M. Daniels, of Buffalo, at the new Buffalo Hospital.

---

THE Homeopathic Medical Society of the State of New York held its semi-annual meeting in Buffalo, September 24-26, 1901. Dr. J. S. Greenleaf, of Owego, is the president, and Dr. D. G. Wilcox, of Buffalo, is the secretary.

---

THE New York State Medical Association held its eighteenth annual meeting at New York, October 21-24, 1901. An excellent program was presented and the meeting, which is still in session at this writing, appears to be a scientific and well attended medical gathering. Physicians from Buffalo in attendance are: Drs. Bernard Cohen, Geo. F. Cott, W. H. Thornton, C. A. Wall, Julius Ullman, A. A. Hubbell, I. P. Lyon, A. T. Lytle, A. G. Bennett, J. F. Krug, F. M. O'Gorman, C. H. Woodward and C. G. Stockton.

---

THE Lake Erie Medical Society held its regular quarterly meeting at Hotel Eggleston, Dayton, N.Y., Friday, October 4, 1901. Papers were presented as follows: Smallpox, William A. Putnam, Silver Creek; The smallpox problem and its difficulties, Ernest Wende, Health Commissioner of Buffalo; The differential diagnosis of infectious diseases, W. M. Ward, North Collins; The differential diagnosis of some eye diseases, Lorenzo Burrows, Buffalo.

